# Two-Way Text Messaging to Support Self-Care and Delivery of an Online Sexual Health Service: Mixed Methods Evaluation

**DOI:** 10.2196/17191

**Published:** 2020-08-20

**Authors:** Sarah Shanks, Alessandra Morelli, Elena Ardines, Gillian Holdsworth, Paula Baraitser

**Affiliations:** 1 SH:24 London United Kingdom; 2 Sexual Health Research Group, King's Centre for Global Health and Health Partnerships, King's College London London United Kingdom; 3 Department of Sexual Health and HIV Kings College Hospital National Health Service Foundation Trust London United Kingdom

**Keywords:** SMS, text message, digital health, sexual health, self-care, mobile phone

## Abstract

**Background:**

Digital health care is increasingly used to improve health service accessibility and reduce costs. Remote health care requires a significant self-management role for service users, and this generates information provision and support needs that should be reflected in service planning. SMS text messaging offers a convenient and low-cost method of communication and is increasingly used across digital health care services to provide remote support.

**Objective:**

The aim of this study was to quantify the number of messages generated through user interaction with a two-way SMS text messaging support service within an online sexual health service and to thematically explore the content of the messages and type of support required to facilitate self-management.

**Methods:**

The content of all SMS text messages received by an online sexual health service was analyzed from April 4, 2018, to July 5, 2018. Messages were classified as being either administrative or clinical in nature and service or user initiated. For those messages that were both clinical and user initiated, a qualitative thematic analysis was completed to fully describe the content of the interactions.

**Results:**

A total of 267 actionable messages were generated per 1000 orders requested through the service. Of the 8562 messages, 5447 (63.62%) messages were administrative and 3115 (36.38%) were clinical. Overall, 4306 of the 8562 messages (50.29%) responded to service-generated queries reflecting the public health and clinical responsibilities of an online provider, and 4256 (49.71%) were user-generated queries, demonstrating a willingness by users to proactively engage with a two-way SMS text messaging support service. Of the 3115 clinical messages, 968 (31.08%) clinical messages were user initiated and shared personal and complex clinical information, including requests for help with the self-testing process and personalized clinical advice relating to symptoms and treatment.

**Conclusions:**

This study demonstrates the willingness of users of an online sexual health service to engage with two-way SMS text messaging and provides insight into the quantity and nature of the support required to facilitate service delivery and self-care. Further work is required to understand the range of clinical problems that can be managed within this medium.

## Introduction

Digital health care is used to improve health service accessibility and reduce costs, and online health services are increasingly part of a “digital first” National Health Service (NHS) [1,2]. Sexual health services have been important innovators in this field, with online testing for sexually transmitted infections (STIs) and online contraception now available in many parts of the United Kingdom.

Digital health services require users to take on new responsibilities for self-care, such as self-taken finger prick blood samples or interpreting results of tests completed at home, and these new roles require additional support [3,4]. This support may be delivered through a wide range of media, including telephone, video conversations, and messaging. Telephone and video conversations were used in early self-care services, possibly because the synchronous and voiced-based elements of these media reproduced face-to-face conversations more closely; however, health services are now progressively using text-based and asynchronous media for remote communications.

SMS text messaging is increasingly used within face-to-face sexual health services to provide test results, information, and support. One-way and two-way text messaging support is convenient, confidential, and anonymous. It is also low cost and accessible, especially to young people [5-9]. One-way messaging (usually service to user) is more commonly used than two-way messaging, and standardized messages appear to have less impact then tailored and customized messages [10]. There have been small-scale pilots of support via text messaging to facilitate self-care within online services [11-13]. However, there has been no evaluation of this type of support when delivered at scale. This gap in the evidence is important, as those providing online services need information about the number and type of messages generated to inform service planning, including staff time and expertise required.

This study reports an analysis of the use of two-way SMS text messaging support within a large online sexual health service. We used both quantitative and qualitative methods to understand (1) the volume of SMS text messages generated through user interaction with the service and (2) the nature of the messages sent by users and type of support required to facilitate service delivery and self-management.

## Methods

### Study Setting

We studied digital communication within a large digital sexual health service based in the United Kingdom. SH:24 is an online sexual health provider that offers self-sampling for STI tests, chlamydia treatment for users who test positive, and online contraception [14]. SH:24 is a community interest company that is commissioned by the NHS to provide services in 35 regions across the United Kingdom. The service was recently expanded to offer Fettle, a paid-for service for people living without a public sector–commissioned online service and who choose to have their care this way. SH:24 was designed to be integrated with face-to-face services and to work as part of the whole system of sexual health care [4], ensuring that users are appropriately referred between online- and clinic-based services according to clinical need.

The SH:24 and Fettle service is provided through an online portal. Users access the portal via a website to order sexual health tests, treatment, or oral contraception. Users are required to submit personal information as part of the ordering process when relevant to the service requested. For example, a sexual behavior history is required for sexual health testing and a medical history is required for contraception orders. Sexual health test kits are delivered to the user’s home, and users take their own samples; this is usually a finger prick blood test, plus a self-taken vaginal swab for women or a urine sample for men. Samples are mailed by users directly to the laboratory and results are provided by SMS or a telephone call, depending on the infection identified. Treatments (for simple chlamydia infection and oral contraceptives) are prescribed based on test results and an online medical history. After prescription by a UK General Medical Council–registered doctor, treatments are dispensed and mailed to users by a UK-registered online pharmacy. The service is available 7 days a week and, at the time of this study, clinical support for the service was provided by a team of 3 specialist sexual health nurses and 1 clinical support worker. The clinical team has since expanded, as demand for the service has increased.

Once an order has been submitted, SMS text messaging is the primary medium for communication between service and user. SMS text messages have multiple functions within the service, from resolving logistical queries between the service and users to providing important health promotion information or guidance about self-management tasks, such as self-sampling or taking treatment. Two-way communication is encouraged and messages end with the option to contact the clinical team for additional information (see Textbox 1). Users can contact the clinical team at any time by replying to a message. Telephone consultations are used to provide reactive HIV results and are offered as part of the risk assessment process for safeguarding concerns.

**Examples of SMS text messages to support self-sampling and promote hepatitis B vaccination for men who have sex with men. GP: general practitioner.**

**Example self-sampling text message**

“Hello. The Royal Mail should deliver your test kit within the next 72hrs. Infections may not show up in tests immediately after exposure, so it's important you test at the right time. If you are taking a blood test, we recommend taking it 4 weeks after your potential exposure - you can watch a 2 minute video to help you take a blood sample here: bit.ly/bloodtestSH24. If you are ONLY testing for chlamydia and gonorrhoea, we recommend taking the test 2 weeks after a potential exposure. Check the best time to take your test here: bit.ly/when2. Text back if you would like help. Thanks, SH:24”

**Example hepatitis B vaccination text message**

“Hello, you have told us that you have not been vaccinated against hepatitis B. Men who have sex with men can get vaccinated for free at any sexual health clinic or GP surgery. Hepatitis B is carried in blood so can be transmitted through having sex with someone who has the virus. Someone who has hepatitis B may appear well, whilst for some people, the infection can be long-lasting and lead to serious liver disease. Having a course of 3 hepatitis B vaccinations is the best way to protect yourself from the infection. Find your local sexual health clinic: https://www.nhs.uk/service-search/other-services/Sexual-health-information-and-support/LocationSearch/734. If you have any questions please reply to this message, your text will be read by a member of our clinical team. Thanks, SH:24”

### Data Collection

We used routinely collected data to describe the demographics of users of SH:24 and Fettle between April 4, 2018, and July 5, 2018. These data are collected every time an order is placed with the service, including when multiple orders are generated by a single user. We extracted the text of all incoming SMS text messages received by SH:24 and Fettle during this time period. The data received contained the content of the message only; there were no details about the sender, their service use, or the time or date of sending. It was not possible to differentiate between text messages sent by users of SH:24 and text messages sent by users of Fettle unless this was specifically referred to in the content of the message.

### Analysis

Demographic data were analyzed using Stata 15 (StataCorp LLC) in terms of age, sex, sexuality, and ethnicity. This analysis was done at the aggregate level to describe the population per order placed, not per individual user.

The SMS messages were analyzed by 3 members of the research team (SS, AM, PB). Messages were categorized by qualitative content and all messages were included in the initial analysis. Through initial reading and rereading of the messages, we developed a simple classification to describe them: (1) administrative or clinical and (2) user generated or service generated.

First, administrative messages were defined as those that required no clinical knowledge by the online support staff to reply to or act on. Clinical messages were defined as all messages that would require clinical knowledge by the online support staff to respond to or act on.

Second, service-generated messages were defined as messages that had been sent by users in response to a question asked by the service. User-generated messages were defined as those with content indicating they had been sent by the user without an initial prompt from the service.

The full data set was then coded according to this system. Messages containing content of no identifiable meaning or purpose were discarded at this stage and excluded from further analysis. The frequencies of different categories of text message were calculated to describe the volume of SMS text messages generated through user interaction with the service and the nature of the messages sent by users.

For messages that were categorized as both clinical and user generated, we completed a qualitative thematic analysis to fully describe the content of the interactions. This involved the research team reading and rereading all messages with an iterative process of theme generation, discussion, and development until a comprehensive structure for describing these data was developed and agreed upon by all the researchers involved in coding (SS, AM, PB). Initial coding frameworks were modified and refined during this process until the team agreed that the final framework effectively reflected the content of the SMS conversations analyzed. The final coding structure included 4 main themes and multiple subthemes. The main themes from the final coding analysis were (1) requests for personal support with the self-management process, (2) requests for help with personal assessment of risk of infection, (3) requests for help interpreting test results, and (4) requests for personalized clinical advice.

A final round of thematic analysis was completed within each category to describe and interpret the material within each main coding theme. When direct quotes were used to illustrate messages, we altered small details of the text to maintain confidentiality without changing the sense or tone of the message.

### Ethics

Approval was obtained as a part of an MSc project from the Health Research Authority Research Ethics Committee (reference: 18/HRA/0128; Integrated Research Application System project ID: 224808).

## Results

### Characteristics of Text Message Service Usage

Between April 4, 2018, and July 5, 2018, there were 38,033 orders requested through the service. Of the 38,033 orders, 34,494 (90.69%) were placed through SH:24 and 3539 (9.31%) through Fettle. The demographics of the users who created the orders are shown in Table 1.

**Table 1:** User demographics for orders requested between April 4, 2018, and July 5, 2018 (N=38,033).

Characteristic			Orders, n (%)
**Age group (years)**			
	16-17	749 (1.97)	
	18-24	16,477 (43.32)	
	25-34	15,316 (40.27)	
	35-54	5077 (13.35)	
	55+	414 (1.09)	
**Gender**			
	Female	23,033 (60.56)	
	Male	15,000 (39.44)	
**Ethnicity**			
	White	24,402 (64.16)	
	Black/African/Caribbean/Black British	3929 (10.33)	
	Asian/Asian British	969 (2.55)	
	Mixed/multiple ethnicity	2172 (5.71)	
	Other	2560 (6.73)	
	Not known/prefer not to say	462 (1.21)	
	Not asked	3539 (9.31)	
**Sexual preference of female users**			
	Men and women	1046 (4.54)	
	Men	21,499 (93.34)	
	Women	488 (2.12)	
**Sexual preference of male users**			
	Men and women	750 (5.00)	
	Men	2584 (17.23)	
	Women	11,666 (77.77)	

**Figure 1:**
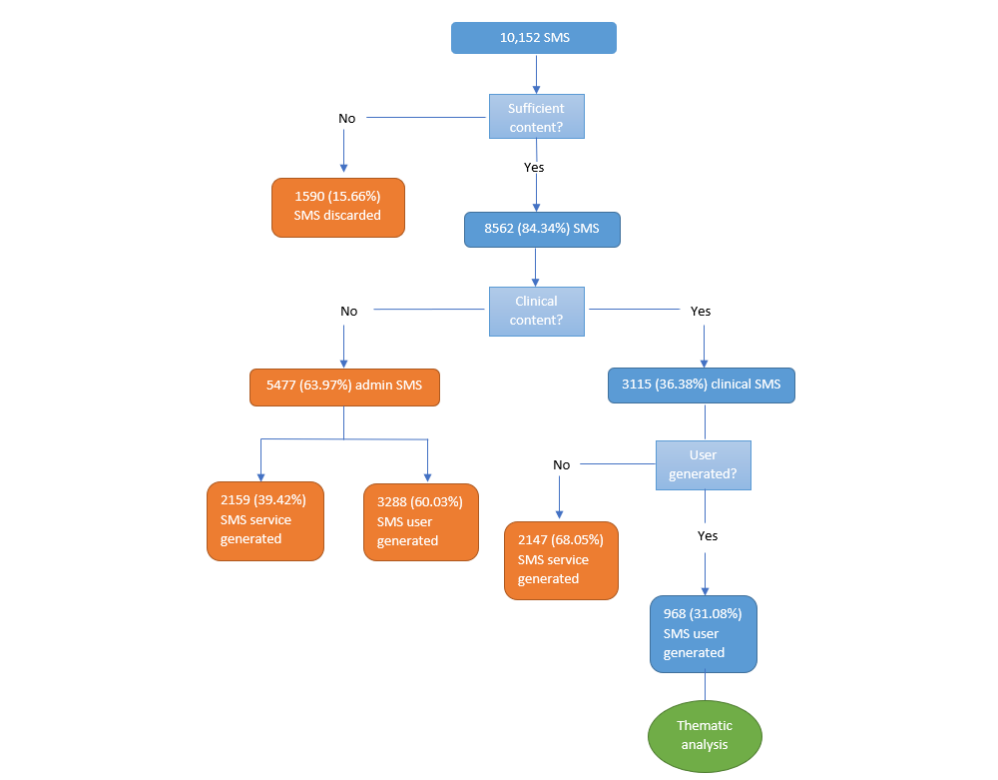
Results of quantitative analysis.

A total of 10,152 incoming SMS text messages were received during the 3-month time period. After the initial analysis, 1590 messages were discarded due to insufficient content. This included messages containing characters or words that did not make grammatical sense, for example “;;; I,” and those that consisted only of generic conversational words or phrases, such as “thank you,” “hello,” or “ok.” Of the remaining 8562 SMS text messages, 5447 (63.62%) were classified as administrative and 3115 (36.38%) were classified as clinical (Figure 1).

Of the 5447 administrative messages, 2159 (39.64%) were service generated to facilitate the running of the service, for example messages confirming user age or address and messages sent in reply to queries from the support team about unused test kits:

Yes that is the correct address.

Yes sorry, hectic time recently!! Will send it off tomorrow, thanks.

A total of 3288 of the 5447 administrative messages (60.36%) were user generated. The majority of these contained logistical queries about user orders, typical of those asked for any online order service:

HI I posted my kit today when do I get results?

Can I track delivery and will I need to sign for it. And will “fettle” appear on packaging?

There were also queries about the services provided at SH:24 and messages from users wanting help navigating sexual health services:

Do you do pregnancy tests?

Can you tell my blood type?

I visited the clinic before and they told me just to get a kit, that was couple of months ago now so I'm a bit hesitant about attending and being told the same thing again.

Hi there, could you please recommend a clinic that can do an instant HIV test?

Of the 3115 messages that were classified as clinical messages, 2147 responded to service-generated questions that reflect the public health role and clinical function of the service. This included messages confirming that partner notification for STIs had been completed or that treatment had been received and taken and messages justifying a request to retest soon after a previous positive test result:

Hello, yes I confirm I have taken the treatment and experienced no vomiting or diarrhoea.

Hi I have had one but am still getting the symptoms. Is it not possible to do one more? The wait to go to the clinic is weeks. Thank you.

A total of 190 messages in this category contained responses to service-generated clinical questions prior to prescribing oral contraception or antibiotics:

Hi, I have been religiously taking my pill and am definitely not pregnant.

Finally, there were a very small number of messages, a total of 24, sent in relation to a safeguarding risk assessment that is completed during the online order process as part of the service’s responsibility to identify and support vulnerable users, particularly those younger than 18 years [15]. In these messages, users were willing to disclose highly sensitive personal information and were open to discussions about support through messaging:

I got forced to have sex.

Hi. I'm not really sure what type of counselling I require, just feel sad and like I am losing interest in things.

### Thematic Analysis of User-Initiated Clinical Messages

A total of 968 out of 3115 messages in the clinical category (31.08%) were user initiated, and an in-depth thematic analysis was completed on these data. The messages in this category had an informal, conversational tone and shared personal, often complex information. The thematic content analysis of these messages identified 4 main subjects of requests for personal support: help with the self-management of STI testing, help to assess risk of infection, requests for support with interpreting test results, and requests for personalized clinical advice.

#### Requests for Personal Support With the Self-Management Process

Messages in this theme were sent by users with questions about STI test self-sampling completion. These included procedural and technical questions about using the kit:

How far do I insert the swab?

Hi, I got a test kit at the beginning of the year but never got round to doing it, can I do it now?

The messages associated with blood test completion described the difficulty that some users had completing self-sampling and the new thinking that the process of a self-taken blood sample generated:

Hi, I've just done the blood collection for the hiv sample, and it took me a long time to fill the tube…my blood is so thick and wouldn't flow out at all. I got a bit worried, isn't it supposed to be more fluid and liquid? Thx for letting me know something.

Hi. Can I only send vaginal swap sample? I fainted when trying to take blood sample so I think it would be better to it done at the clinic. Thanks?

#### Requests for Personal Assessment of Risk of Infection

These messages reflected the level of concern generated by the possibility of STI transmission and the work that users did to predict their chances of infection, even during the process of testing. In these situations, complex descriptions were provided to support the anticipated clinical assessment of their risk:

So I received oral sex from male that has chlamydia but he contracted it through genital sex. He didn’t give oral sex to the person he contracted it from and when I received oral sex from him no fluids except saliva were exchanged. Is there any chance that I could receive chlamydia?

#### Help With Interpreting Test Results

Many users required reassurance about the accuracy and reliability of the tests when they received an unexpected result:

How accurate are these tests…My boyfriend tested positive and got treatment. So i got tested with yourselfs and the test I received the results from showed negative?! This makes no sense?

Thank you, is that 100%? Or could I just not have swabbed enough?

Other users needed support to understand the actual meaning of the test results:

To clarify: my result was “negative”. Does this mean I'm 100% sure about it being negative and there is no need to repeat any test? Thanks.

#### Requests for Personalized Clinical Advice

Messages in this theme contained clinical information that required a decision and response by a skilled health care professional. The messages were mainly related to the more complex services provided online (antibiotic treatment and contraception) or to users experiencing symptoms of infection:

I have itching which I thought was thrush- would this need additional treatment?

Hi I have white patches around my area down there, there was a lot more than there is right now but I've looked at them with a mirror and I'm really worried now.

The answers to these queries would not be available on general sexual health information websites and related to specific, often complex, clinical circumstances.

## Discussion

### Principal Findings

This study provides insight into how people interact with a two-way SMS text messaging support service within an online sexual health service and demonstrates the quantity and nature of support required to administer the service and facilitate self-care by users. During the study period, 8562 actionable SMS messages were received, corresponding to 267 messages generated per 1000 orders requested. A total of 63.62% (5447/8562) of the messages were administrative and 36.38% (3115/8562) required action by a member of staff with clinical knowledge.

Overall, 4306 of the 8562 actionable messages (50.29%) responded to service-generated queries that reflect the public health and clinical responsibilities of this service, and 4256 (49.71%) were user-initiated queries, demonstrating a willingness by users to proactively engage with a two-way SMS text messaging support service. In-depth analysis of the user-generated clinical messages was completed to explore the complexity of clinical questions asked by users over SMS. The results demonstrated the acceptability of providing personal information and discussing sensitive clinical matters using this platform. The queries highlighted the gaps in skills (for self-taken samples) and knowledge (to manage unanticipated issues such as medication side effects) that are needed to complete the self-care process.

The use of SMS to support personalized clinical communication is part of a shift from remote communication by synchronous and voice-based communication media, such as telephone or video, to asynchronous and text-based media, such as SMS text messaging. This option may be less intrusive or susceptible to interruptions, more durable, and less likely to be subject to distortion than voice-based alternatives, and it reflects a change in the usage landscape of mobile devices [16,17]. The level of SMS support required is significant and needs adequate resourcing, and this should be built into new online service development. Since the time this study was completed, the combined SH:24 and Fettle service has expanded from processing 38,000 orders in a 3-month period to a current average of approximately 25,000 orders per month. The findings of the study were used to inform service planning, as the demand for online services increased. It is our experience that it takes an average of 2 minutes to act on an SMS text message. We calculate staff time per orders on the assumption that 25% of user orders (across STI kits, treatment, and contraception) will generate an incoming text message, based on the results of this study. For example, 25,000 orders will require staff to answer 6250 messages. We assume that 60% of the messages will require administrative action and 40% will require a response from a clinically trained member of staff. Therefore, 25,000 orders translates to 125 hours of administrator time and 83.3 hours of clinical time. These calculations reflect time spent on the dedicated SMS text messaging support service only and do not include the other clinical and administrative tasks required in a service of this kind, such as telephone and email communications, prescribing, and supporting partner notification.

Text messaging solutions for health care have been successfully adopted to assist with remote clinical monitoring, information and education services, adherence to treatment or self-management, and consultation [18]. We conclude that the “digital first” plans for health care delivery would benefit from a better understanding of the value of this type of support. Further work is required to understand the range of clinical problems that can be managed within this medium outside of sexual health services.

### Limitations

The main limitation of this study is that text messages could not be identified by service user. This information would determine what proportion of users engaged with the SMS support service and whether there were differences between users of a free or paid-for service. It would also enable patterns of communication between service providers and individual users to be examined. This information would not have altered the findings of the current study; the relative amounts of administrative and clinical support required for the online service is dependent on the content and number of messages received, not on the number of users responsible for sending them. However, further studies could provide additional useful insight into the volume and nature of user interactions with online health services. The generalizability of our findings will be variable across contexts depending on norms of SMS text messaging use and stigma surrounding the discussion of sexual health.

## Abbreviations

NHS: National Health Service
STI: sexually transmitted infection
